# Supplementary material for: The Neurotrophic Function of Glucagon-Like Peptide-1 Promotes Human Neuroblastoma Differentiation via the PI3K-AKT Axis
Source: Biology (Basel). 2020 Oct 22;9(11):348. doi: 10.3390/biology9110348 (PMC7690389; doi:10.3390/biology9110348)

Fig1c-GLP1R 53KDa

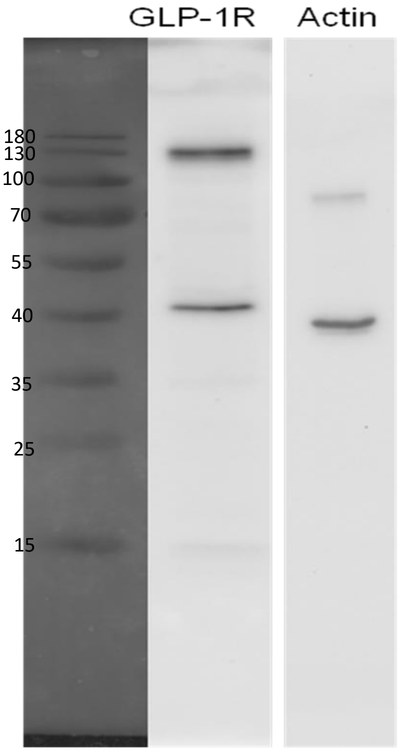

Fig 3a-Vim 57KDa

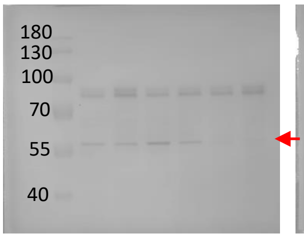

Fig3a-β-actin 42KDa

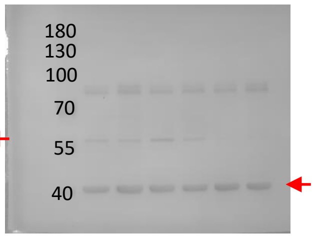

Fig3b-AMPAR 100KDa

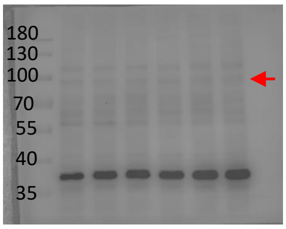

Fig3b-β-actin 42KDa

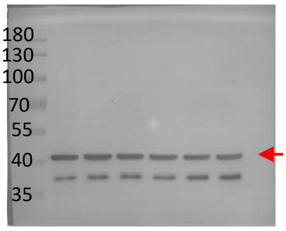

Fig3c-NMDAR 180KDa

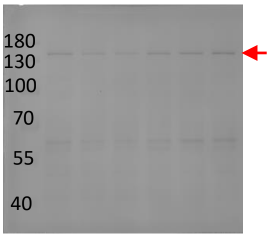

Fig3c-β-actin 42KDa

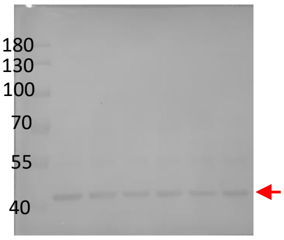

Fig3d-D1R 49KDa

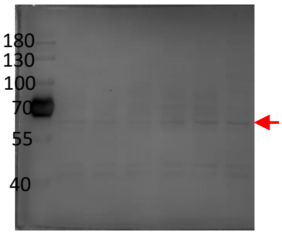

Fig3d-β-actin 42KDa

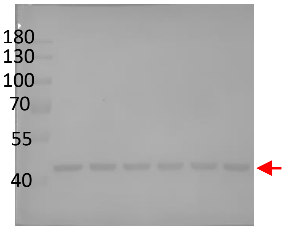

Fig3e-nAChR 70KDa

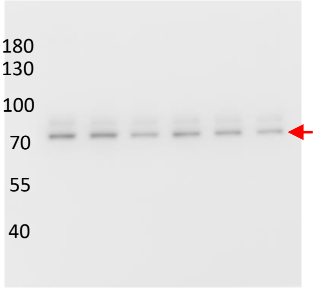

Fig3e-β-actin 42KDa

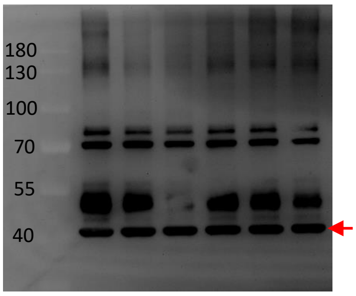

Fig3f-mAChR 52KDa

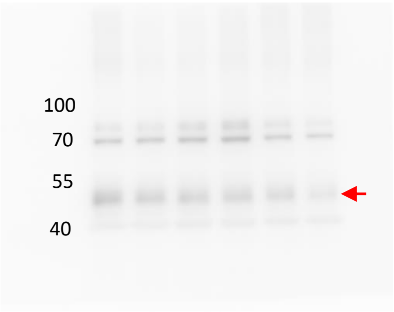

Fig3f-β-actin 42KDa

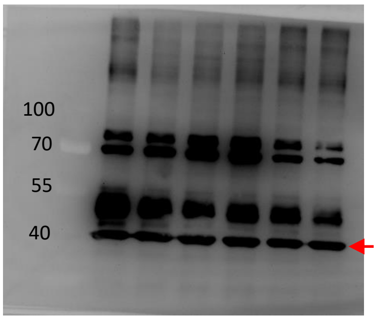

Fig4a-Synapsin 1 77KDa

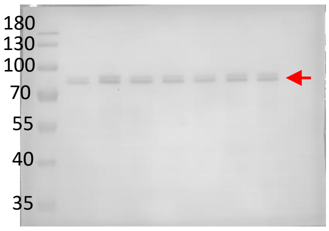

Fig4a-β-actin 42KDa

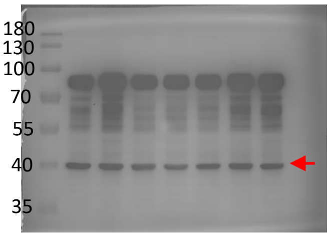

Fig4b-Synaptophysin 40KDa

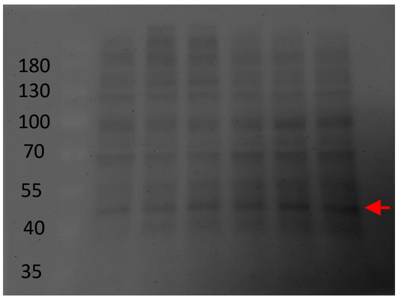

Fig4b-β-tubulin 55KDa

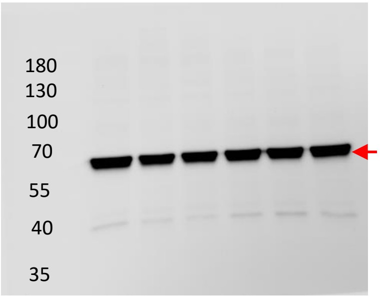

Fig4c-PSD95 95KDa

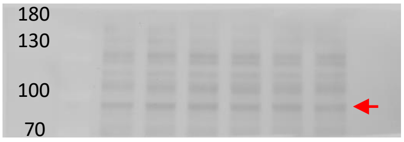

Fig4b-β-tubulin 55KDa

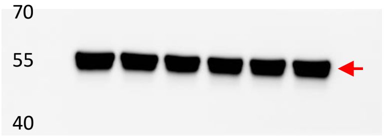

Fig4d-AChE 71KDa

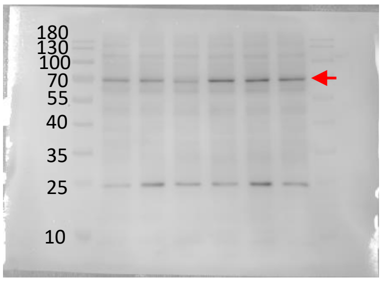

Fig4d-β-actin 42KDa

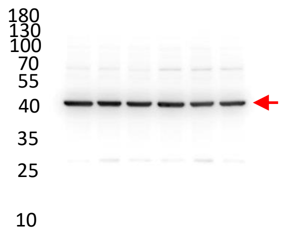

Supplement: Supplementary file 1 [file biology-09-00348-s001.pdf]
